# Efficacy of Guided Imagery Training in Improving Sleep Quality in Patients With Chronic Obstructive Pulmonary Disease and Sleep Disorders

**DOI:** 10.62641/aep.2258

**Published:** 2026-10-15

**Authors:** Qian Ren, Haiyan Zhang, Xiaofen Shi, Lingyan Ren, Jing Cheng

**Affiliations:** ^1^College of Nursing, Yangzhou University, 225009 Yangzhou, Jiangsu, China; ^2^Department of Infectious Diseases, The Affiliated Wuxi People's Hospital of Nanjing Medical University (Wuxi Medical Center, Nanjing Medical University, Wuxi People's Hospital), 214000 Wuxi, Jiangsu, China; ^3^Internal Medicine Department, The Affiliated Wuxi People's Hospital of Nanjing Medical University (Wuxi Medical Center, Nanjing Medical University, Wuxi People's Hospital), 214000 Wuxi, Jiangsu, China; ^4^Nursing Department, Wuxi Huishan District People's Hospital, 214000 Wuxi, Jiangsu, China

**Keywords:** chronic obstructive pulmonary disease, sleep disorders, guided imagery training, sleep quality, Pittsburgh Sleep Quality Index

## Abstract

**Background:**

This work aimed to investigate the effectiveness of guided imagery training in improving sleep quality for patients with chronic obstructive pulmonary disease (COPD) and sleep disorders.

**Methods:**

This retrospective cohort study utilised propensity score matching (PSM). Patients with stable COPD complicated by sleep disorders, treated at The Affiliated Wuxi People’s Hospital of Nanjing Medical University from March 2024 to June 2025, were consecutively identified through a retrospective review of medical records. Sixty patients who underwent guided imagery training combined with standard pharmacotherapy were assigned to the combined treatment group, and 109 patients who received standard pharmacotherapy alone were assigned to the control group. PSM (1:1 nearest neighbour matching without replacement, calliper = 0.02) was applied to balance the two groups, resulting in 53 patients per group for final analysis. The primary outcomes were sleep architecture parameters (e.g., sleep efficiency) and the Pittsburgh Sleep Quality Index (PSQI) score. Secondary outcomes included the Athens Insomnia Scale (AIS) score and pulmonary function indicators. Differences in sleep architecture parameters, AIS scores and PSQI scores were compared between the two groups before and after treatment.

**Results:**

Before treatment, no statistically significant differences were observed between the two groups in terms of sleep efficiency, sleep latency, number of awakenings, apnoea–hypopnea index (AHI) or minimum oxygen saturation (*p* > 0.05). After treatment, both groups showed significant improvements, including increased sleep efficiency and minimum oxygen saturation (*p* < 0.05) and decreased sleep latency, number of awakenings and AHI (*p* < 0.05), compared with baseline. Post-treatment, the combined treatment group demonstrated significantly higher sleep efficiency and minimum oxygen saturation and significantly lower sleep latency, number of awakenings and AHI than the control group (*p* < 0.05). Regarding AIS and PSQI scores, no significant intergroup differences were found at baseline (*p* > 0.05). After treatment, both groups exhibited significant score reductions (*p* < 0.05), with the combined treatment group achieving significantly lower scores than the control group (*p* < 0.05).

**Conclusions:**

In this retrospective cohort of patients with COPD and sleep disorders, the addition of guided imagery training to the standard pharmacotherapy was associated with significant improvements in subjective and objective sleep quality parameters. This approach may serve as a beneficial adjunctive therapy in this population.

## Introduction

Chronic obstructive pulmonary disease (COPD) is a chronic airway disease characterised by persistent airflow limitation, manifesting as cough, sputum production, shortness of breath, wheezing, chest tightness and dyspnoea. Sleep disorders, including difficulty initiating sleep and frequent nocturnal awakenings, are common comorbidities in patients with COPD. These are often attributed to factors such as progressive decline in respiratory function, nocturnal hypoxia, dyspnoea and anxiety or depression [1]. Currently, no standardised treatment protocol is available for COPD-associated sleep disorders. Clinical management typically involves bronchodilators, corticosteroids and oxygen therapy, sometimes supplemented with short-term, low-dose sedative-hypnotics. However, the overall efficacy is often suboptimal, and potential adverse drug reactions can add to patient discomfort [2]. Research suggests that guided imagery training may hold value in treating post-stroke sleep disorders, improving sleep quality and promoting recovery [3]. However, studies investigating its application in patients with COPD complicated with sleep disorders are scarce, thus limiting its clinical guidance. Although cognitive behavioural therapy for insomnia (CBT-I) is the first-line non-pharmacological treatment, its accessibility in respiratory wards may be limited by resource availability and patient tolerance [4]. Guided imagery training, as a simpler, low-cost and easily implemented relaxation technique, warrants investigation as a potential alternative or adjunctive therapy [5]. The present study aims to evaluate the association between guided imagery training and sleep quality in this patient population to provide evidence for clinical practice.

## Materials and Methods

### General Information

This retrospective cohort study was based on a review of medical records. Given that treatment allocation was determined by clinical judgment rather than randomisation, this study was not prospectively registered in a clinical trial registry. Patients with stable COPD complicated by sleep disorders, who were treated at The Affiliated Wuxi People’s Hospital of Nanjing Medical University from March 2024 to June 2025, were consecutively identified through a retrospective review of electronic medical records. The patient screening process is illustrated in Fig. 1. A total of 988 patients with stable COPD and comorbid sleep disorders were initially screened during the study period. After the prespecified exclusion criteria were applied, 819 patients were excluded, leaving 169 patients eligible for inclusion before matching. Of these, 60 patients who underwent guided imagery training combined with standard pharmacotherapy constituted the combined treatment group, and 109 patients who received standard pharmacotherapy alone formed the control group. Propensity score matching (PSM) was employed to minimise selection bias and simulate randomisation effects. A multivariable logistic regression model was used to estimate propensity scores, with the following baseline covariates: age, body mass index (BMI), Pittsburgh Sleep Quality Index (PSQI) score, COPD duration, insomnia duration, heart rate (HR), respiratory rate (RR), Global Initiative for Chronic Obstructive Lung Disease (GOLD) grade, sex, smoking, alcohol consumption, hypertension, diabetes, hyperlipidaemia and coronary heart disease. A 1:1 nearest neighbour matching algorithm was applied without replacement, using a calliper width of 0.02. After matching, 53 patients were successfully assigned to each group, yielding a final cohort of 106 patients. The two groups were well-balanced on all matching variables (*p* > 0.05). This study was approved by the Medical Ethics Expert Committee of The Affiliated Wuxi People’s Hospital of Nanjing Medical University (Ethics Approval Number: KY26027) and conducted in accordance with the Declaration of Helsinki. Informed consent was obtained from all patients, and data confidentiality was maintained per medical ethics requirements.

**Fig. 1.**
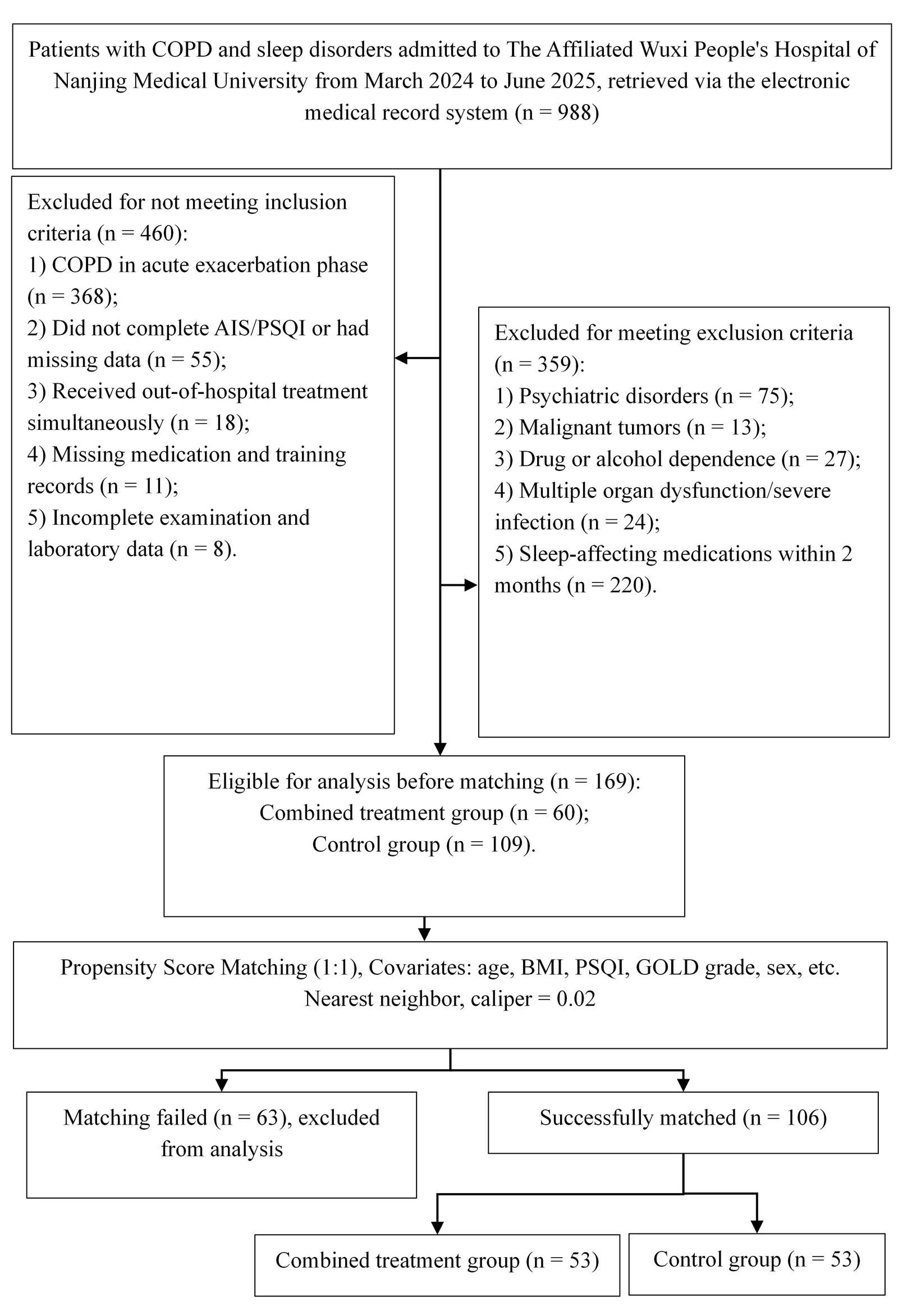
**Flowchart of sample selection**. COPD, chronic obstructive pulmonary disease; BMI, body mass index; PSQI, Pittsburgh Sleep Quality Index; GOLD, Global Initiative for Chronic Obstructive Lung Disease; AIS, Athens Insomnia Scale.

Diagnostic criteria: (1) COPD diagnosis based on the “Global Strategy for the Diagnosis, Management, and Prevention of Chronic Obstructive Lung Disease” [6]; and (2) sleep disorder diagnosis based on the “International Classification of Sleep Disorders, Third Edition” [7], with a PSQI score ≥7, a widely used cutoff indicating poor sleep quality in clinical populations [8].

Inclusion criteria: (1) age ≥18 years; (2) COPD in stable phase; (3) completed full course of treatment at our hospital; (4) complete medical records, including completed Athens Insomnia Scale (AIS) and PSQI questionnaires; (5) documented adherence to prescribed medication and guided imagery sessions (where applicable) as per nursing records; and (6) complete examination and laboratory data.

Exclusion criteria: (1) history of psychiatric disorders (e.g., depression, anxiety, bipolar disorder and schizophrenia) or significant mood disturbance noted in medical records; (2) malignant tumours; (3) drug or alcohol dependence; (4) multiple organ dysfunction or severe infection during hospitalization; (5) use of sleep-affecting medications within 2 months prior to enrolment, as documented in medication history; and (6) shift work status.

### Standard Pharmacotherapy

The control group received standard pharmacotherapy alone, including the following: budesonide formoterol inhaled powder II (AstraZeneca Pharmaceuticals, specification: 320 μg of budesonide and 9.0 μg of formoterol fumarate per inhalation, National Drug Approval Number: HJ20160447), one inhalation per dose, twice daily [9]; and eszopiclone (Changzhou Siyao, specification: 3 mg, National Drug Approval Number: H20213536), 1–3 mg once daily before bedtime. Efficacy was evaluated after 4 weeks of treatment [10].

### Guided Imagery Training

Guided Imagery Training [11]: The combined treatment group received guided imagery training in addition to the standard pharmacotherapy described above. The personnel conducting the training were qualified physicians (attending or associate chief physician level) in psychiatry who had undergone standardised training in psychological therapy. Training sessions were conducted in a quiet, dimly lit and comfortably warm room with a “Do Not Disturb” sign. A standardised script framework was adapted to individual patient needs and preferences to ensure consistency whilst allowing personalisation.

The patients were instructed to assume a comfortable seated or supine position, relax completely, close their eyes and establish a steady breathing rhythm by using pursed-lip or abdominal breathing. A therapist used a slow, gentle voice to guide the patients in constructing vivid mental images (e.g., meadows, hillsides and seashores), engaging all five senses, accompanied by natural sounds like bird songs or flowing water. The patients were guided to imagine themselves walking freely in these scenes, breathing fresh air flowing gently into their lungs. The sessions lasted 15 min, and they were conducted once daily. Adherence was monitored by nursing staff who recorded session completion in the medical record after each session. A researcher verified these records weekly to ensure fidelity to the training protocol. Efficacy was evaluated after 4 weeks of treatment.

### Outcome Measures

Primary and secondary outcomes were pre-specified. The primary outcomes were sleep architecture parameters [sleep efficiency, sleep latency, number of awakenings, apnoea–hypopnea index (AHI) and minimum oxygen saturation] measured by polysomnography, and the global PSQI score. The secondary outcomes were the AIS score and pulmonary function indicators. All data were extracted from the medical records before and after the 4-week treatment period.

AIS [12] was administered before and after treatment. This scale assesses eight items: sleep onset, nighttime awakening, early morning awakening, total sleep time, overall sleep quality, daytime mood, daytime physical/mental function and daytime sleepiness. Each item is scored 0–3, with a maximum total score of 24. Higher scores indicate more severe sleep disturbance. AIS has been validated in Chinese populations and demonstrated good reliability in the present study sample (Cronbach’s α = 0.855).

PSQI [13] was administered before and after treatment. It comprises seven component scores: subjective sleep quality, sleep latency, sleep duration, sleep efficiency, sleep disturbances, use of sleep medication and daytime dysfunction. Each component is scored 0–3, with a maximum global score of 21. Higher scores indicate poorer sleep quality. PSQI has been validated in Chinese populations and demonstrated good reliability in the present study sample (Cronbach’s α = 0.881).

Pulmonary function indicators, including forced expiratory volume in one second as a percentage of predicted (FEV1%pred) and the ratio of forced expiratory volume in one second to forced vital capacity (FEV1/FVC), were measured before and after treatment by using a spirometer (Shenzhen Mindray Bio-Medical Electronics Co., Ltd., Model MasterScreen).

### Statistical Analysis

All data were analysed using R software (version 4.0.0; R Foundation for Statistical Computing, Vienna, Austria). PSM was employed to minimise selection bias. Propensity scores were estimated using a multivariable logistic regression model that included the following baseline covariates: age, BMI, PSQI score, gender and GOLD grade. A 1:1 nearest neighbour matching algorithm was applied without replacement, using a calliper width of 0.02. The normality of continuous variables was assessed using the Shapiro–Wilk test. On the basis of the results of normality assessment, normally distributed continuous variables were presented as mean ± standard deviation and compared using independent sample *t*-test for between-group comparisons and paired *t*-test for within-group comparisons. Non-normally distributed continuous variables were presented as median and interquartile range and were compared using the Mann–Whitney U test for between-group comparisons and the Wilcoxon signed-rank test for within-group comparisons. If all variables in a given analysis were normally distributed and no non-normal variables were present, the parametric tests described above were applied uniformly as stated. Categorical variables were presented as frequencies and percentages and compared using chi-square (χ^2^) test or Fisher’s exact test, as appropriate. A Bonferroni correction was applied to account for multiple comparisons across several endpoints. A two-sided *p*-value < 0.05 was considered statistically significant. The balance of covariates after matching was assessed using standardised mean differences (SMDs), with an SMD < 0.1 considered indicative of good balance.

## Results

### Comparison of General Data Before and After PSM

Before PSM, no statistically significant differences were found between the combined treatment and control groups in terms of BMI, COPD duration, insomnia duration, HR, RR, smoking, alcohol consumption or comorbidities (hypertension, diabetes, hyperlipidaemia and coronary heart disease; *p* > 0.05). However, significant differences existed in age, PSQI score, GOLD grade and gender distribution (*p* < 0.05), as shown in Table 1.

**Table 1.** **Comparison of general data between the combined treatment and control groups before PSM**.

Characteristic		Before PSM			
		Combined treatment group (n = 60)	Control group (n = 109)	*t*/χ^2^	*p*
Age (years)		65.11 ± 7.43	62.81 ± 6.60	2.072	0.040
BMI (kg/m^2^)		23.47 ± 1.90	23.60 ± 2.13	–0.394	0.694
COPD duration (years)		8.93 ± 2.33	8.40 ± 3.01	1.182	0.239
Insomnia duration (years)		23.91 ± 4.88	22.83 ± 5.50	1.270	0.206
HR (beats/min)		86.93 ± 8.10	88.04 ± 7.46	–0.898	0.371
RR (beats/min)		23.37 ± 1.85	23.01 ± 1.92	1.181	0.239
PSQI score		13.61 ± 2.40	12.04 ± 2.51	3.951	<0.001
Gender				6.894	0.009
	Male	34 (56.67)	83 (76.15)		
	Female	26 (43.33)	26 (23.85)		
GOLD grade, n (%)				5.155	0.023
	Grade 2	21 (35)	58 (53.21)		
	Grade 3	39 (65)	51 (46.79)		
Smoking, n (%)				1.498	0.221
	Yes	18 (30)	43 (39.45)		
	No	42 (70)	66 (60.55)		
Alcohol, n (%)				0.839	0.360
	Yes	21 (35)	46 (42.2)		
	No	39 (65)	63 (57.8)		
Hypertension, n (%)				0.471	0.493
	Yes	21 (35)	44 (40.37)		
	No	39 (65)	65 (59.63)		
Diabetes, n (%)				1.76	0.185
	Yes	12 (20)	32 (29.36)		
	No	48 (80)	77 (70.64)		
Hyperlipidaemia, n (%)				0.462	0.497
	Yes	27 (45)	55 (50.46)		
	No	33 (55)	54 (49.54)		
Coronary heart disease, n (%)				0.925	0.336
	Yes	11 (18.33)	14 (12.84)		
	No	49 (81.67)	95 (87.16)		

Note: PSM, propensity score matching; BMI, body mass index; COPD, chronic obstructive pulmonary disease; HR, heart rate; RR, respiratory rate; PSQI, Pittsburgh Sleep Quality Index; GOLD, Global Initiative for Chronic Obstructive Lung Disease.

After PSM, the combined treatment and control groups were well-balanced, with no statistically significant differences in any baseline characteristics (all *p* > 0.05). Additionally, the SMDs for all covariates were <0.1, confirming adequate balance (Table 2).

**Table 2.** **Comparison of general data between the combined treatment and control groups after PSM**.

Characteristic		After PSM				
		Combined treatment group (n = 53)	Control group (n = 53)	SMD	*t*/χ^2^	*p*
Age (years)		63.81 ± 7.55	62.55 ± 6.60	0.07	0.915	0.362
BMI (kg/m^2^)		23.80 ± 1.90	23.54 ± 2.13	0.09	0.663	0.509
COPD duration (years)		8.77 ± 2.16	8.16 ± 2.85	0.07	1.242	0.217
Insomnia duration (years)		23.20 ± 5.10	22.75 ± 4.96	0.06	0.460	0.646
HR(beats/min)		87.17 ± 6.95	86.63 ± 7.22	0.05	0.392	0.696
RR(beats/min)		23.14 ± 1.67	23.56 ± 1.70	0.08	–1.283	0.202
PSQI score		12.86 ± 2.26	12.43 ± 2.27	0.03	0.977	0.331
Gender					0.614	0.433
	Male	28 (52.83)	32 (60.38)			
	Female	25 (47.17)	21 (39.62)			
GOLD grade, n (%)					0.656	0.418
	Grade 2	17 (32.08)	21 (39.62)			
	Grade 3	36 (67.92)	32 (60.38)			
Smoking, n (%)					1.477	0.224
	Yes	16 (30.19)	22 (41.51)			
	No	37 (69.81)	31 (58.49)			
Alcohol, n (%)					0.642	0.423
	Yes	18 (33.96)	22 (41.51)			
	No	35 (66.04)	31 (58.49)			
Hypertension, n (%)					1.514	0.218
	Yes	15 (28.3)	21 (39.62)			
	No	38 (71.7)	32 (60.38)			
Diabetes, n (%)					1.835	0.173
	Yes	10 (18.87)	16 (30.19)			
	No	43 (81.13)	37 (69.81)			
Hyperlipidaemia, n (%)					0.614	0.433
	Yes	21 (39.62)	25 (47.17)			
	No	32 (60.38)	28 (52.83)			
Coronary heart disease, n (%)					0.577	0.447
	Yes	8 (15.09)	11 (20.75)			
	No	45 (84.91)	42 (79.25)			

Note: An SMD < 0.1 indicates good balance between groups. BMI, body mass index; COPD, chronic obstructive pulmonary disease; HR, heart rate; RR, respiratory rate; PSQI, Pittsburgh Sleep Quality Index; GOLD, Global Initiative for Chronic Obstructive Lung Disease; PSM, propensity score matching; SMD, standardised mean difference.

### Comparison of Sleep Architecture Parameters After PSM

Before treatment, no significant differences were found between the two groups in terms of sleep efficiency, sleep latency, number of awakenings, AHI and minimum oxygen saturation (*p* > 0.05). After treatment, both groups showed significant improvements compared with baseline: increased sleep efficiency and minimum oxygen saturation (*p* < 0.05) and decreased sleep latency, number of awakenings and AHI (*p* < 0.05). Post-treatment, the combined treatment group exhibited significantly better outcomes than the control group, with higher sleep efficiency and minimum oxygen saturation and lower sleep latency, number of awakenings and AHI (*p* < 0.05, Table 3).

**Table 3.** **Comparison of sleep architecture parameters between the two groups after PSM**.

Sleep architecture parameters	Time	Combined treatment group (n = 53)	Control group (n = 53)	*t*	*p*
Sleep efficiency (%)	Pre-treatment	61.54 ± 4.91	62.50 ± 4.54	–1.045	0.298
	Post-treatment	78.58 ± 5.55*	74.16 ± 5.76*	4.023	<0.001
Sleep latency (min)	Pre-treatment	43.10 ± 5.96	45.12 ± 4.65	–1.945	0.054
	Post-treatment	24.17 ± 3.30*	28.40 ± 4.32*	–5.665	<0.001
Awakenings (times)	Pre-treatment	4.58 ± 1.50	4.16 ± 1.74	1.331	0.186
	Post-treatment	1.95 ± 0.77*	2.65 ± 0.82*	–4.530	<0.001
AHI (events/h)	Pre-treatment	14.38 ± 2.80	13.90 ± 3.10	0.837	0.405
	Post-treatment	9.46 ± 1.86*	11.04 ± 2.37*	–3.818	<0.001
Minimum O_2_ saturation (%)	Pre-treatment	82.47 ± 4.18	83.55 ± 4.56	–1.271	0.207
	Post-treatment	90.43 ± 4.75*	87.17 ± 3.48*	4.031	<0.001

Note: PSM, propensity score matching; AHI, apnoea–hypopnea index. *p < 0.05 compared with pre-treatment within the same group (paired *t*-test). *p*-values for between-group comparisons are from independent sample *t*-tests with Bonferroni correction.

### Comparison of AIS and PSQI Scores After PSM

Before treatment, no significant differences were observed between the two groups in terms of AIS and PSQI scores (*p* > 0.05). After treatment, both groups demonstrated significant score reductions compared with baseline (*p* < 0.05). The combined treatment group achieved significantly lower post-treatment AIS and PSQI scores than the control group (*p* < 0.05, Table 4).

**Table 4.** **Comparison of AIS and PSQI scores between the two groups after PSM**.

Parameters	Time	Combined treatment group (n = 53)	Control group (n = 53)	*t*	*p*
AIS (point)	Pre-treatment	15.38 ± 2.96	14.73 ± 2.78	1.165	0.247
	Post-treatment	8.15 ± 2.30*	9.76 ± 2.43*	–3.503	0.001
PSQI score (point)	Pre-treatment	12.86 ± 2.26	12.43 ± 2.27	0.977	0.331
	Post-treatment	6.17 ± 1.80*	8.22 ± 2.03*	–5.501	<0.001

Note: PSQI, Pittsburgh Sleep Quality Index; AIS, Athens Insomnia Scale; PSM, propensity score matching. *p < 0.05 compared with pre-treatment within the same group (paired *t*-test). *p*-values for between-group comparisons are from independent samples *t*-tests with Bonferroni correction.

### Comparison of Pulmonary Function Indicators After PSM

Before treatment, no significant differences were found between the two groups in terms of FEV1%pred and FEV1/FVC (*p* > 0.05). After treatment, neither group showed significant changes in these parameters compared with baseline (*p* > 0.05). Furthermore, no significant intergroup differences were observed in post-treatment FEV1%pred or FEV1/FVC (*p* > 0.05), as shown in Table 5.

**Table 5.** **Comparison of pulmonary function indicators between the two groups after PSM**.

Parameters	Time	Combined treatment group (n = 53)	Control group (n = 53)	*t*	*p*
FEV1%pred (%)	Pre-treatment	51.38 ± 4.10	52.30 ± 4.67	–1.078	0.284
	Post-treatment	53.20 ± 4.63	53.87 ± 4.74	–0.736	0.463
FEV1/FVC (%)	Pre-treatment	57.18 ± 3.96	58.00 ± 4.04	–1.055	0.294
	Post-treatment	58.40 ± 3.74	58.86 ± 4.57	–0.567	0.572

Note: PSM, propensity score matching; FEV1%pred, forced expiratory volume in one second as a percentage of predicted; FEV1/FVC, the ratio of forced expiratory volume in one second to forced vital capacity.

## Discussion

This retrospective analysis investigated the efficacy of guided imagery training as an adjunct to standard pharmacotherapy in patients with COPD and comorbid sleep disorders. The principal findings were that the combined treatment was associated with significantly greater improvements in objective sleep architecture parameters (increased sleep efficiency and reduced sleep latency and awakenings) and subjective sleep quality scores (AIS and PSQI) than pharmacotherapy alone. These findings align with and extend those of previous works investigating relaxation-based techniques in respiratory populations.

Sleep disorders affect a substantial proportion of patients with COPD, with independent risk factors including a history of sleep apnoea, anxiety and prolonged disease duration [14]. Whilst bronchodilators and corticosteroids are cornerstones of management to improve nocturnal ventilation and reduce awakenings [15, 16], their effect on sleep quality per se is often incomplete. Sedative-hypnotics like eszopiclone offer direct sleep-promoting effects but carry concerns regarding long-term dependence and adverse outcomes [17]. In this context, non-pharmacological approaches are of great interest. Whilst CBT-I remains the gold standard, its implementation in busy respiratory clinics faces practical barriers. Guided imagery offers a pragmatic, scalable alternative that can be delivered by trained personnel.

Guided imagery training is an emerging approach in chronic disease management. It involves systematically guiding individuals to construct specific, vivid mental images, thereby regulating emotions, alleviating symptoms, enhancing psychological resilience and fostering a sense of control over one’s body [18]. Chen *et al*. [19] applied guided imagery training in patients with stroke and found it beneficial in reducing sleep disturbances and improving health status. The present study explored its application in patients with COPD complicated with sleep disorders. The results showed that combining guided imagery training with pharmacotherapy significantly reduced AIS and PSQI scores, indicating improved sleep quality. This technique, based on mental imagery, guides patients to construct vivid scenes accompanied by corresponding natural sounds. Guiding patients to imagine themselves within these scenes may inhibit excessive amygdala activity, shifting the nervous system from sympathetic to parasympathetic dominance. This shift induces physical and mental relaxation, allowing the body to enter a reparative state and reducing stress responses [20, 21]. Concurrently, incorporating pursed-lip or abdominal breathing helps maintain positive end-expiratory pressure, supporting collapsed small airways in patients with COPD. This facilitates slow, complete exhalation of residual air, thereby enhancing gas exchange efficiency, reducing the work of breathing and alleviating dyspnoea, potentially leading to decreased nocturnal awakenings [22, 23, 24]. In comparison with other relaxation studies, the magnitude of PSQI reduction observed in the combined treatment group (6.7 points) is comparable to or slightly larger than that reported in some mindfulness-based therapies for chronic insomnia, though direct comparisons are limited by different populations and methodologies.

Given the subjective nature of AIS and PSQI, this study utilised polysomnography to objectively and comprehensively evaluate sleep architecture. The findings revealed that guided imagery training combined with standard pharmacotherapy improved sleep efficiency, shortened sleep latency, reduced awakenings and apnoea–hypopnea events and increased oxygen saturation. Bronchodilators and inhaled corticosteroids help reduce airway inflammation and hyperresponsiveness, improving ventilatory function [25]. Eszopiclone binds to the α subunit of the gamma-aminobutyric acid (GABA) type A receptor, enhancing GABA-mediated chloride influx, leading to neuronal hyperpolarisation, reduced central nervous system excitability, shortened sleep onset time and decreased awakenings [26, 27]. Guided imagery training, by actively guiding the brain towards relaxing mental scenes, activates the parasympathetic nervous system and inhibits sympathetic overactivity. This process can significantly lower HR, blood pressure and muscle tension; reduce stress hormone secretion like cortisol; and facilitate a smooth transition from an “alert state” to a “restorative state”, thus creating a physiological foundation for rapid sleep onset whilst mitigating negative emotions and establishing conditioned sleep reflexes [28, 29, 30]. However, improvements in AHI and minimum oxygen saturation should be interpreted with caution. Guided imagery is primarily a relaxation technique and unlikely to directly alleviate upper airway obstruction. The observed improvements may be indirect, mediated by reduced sleep fragmentation and more stable breathing patterns during consolidated sleep states.

Interestingly, the pulmonary function parameters (FEV1%pred and FEV1/FVC) did not significantly change over the 4-week period in either group. This stability suggests that the combined treatment was not detrimental to lung function and may contribute to maintaining disease stability. However, the short follow-up duration precludes any conclusion regarding long-term preservation of lung function, thereby warranting investigation in studies with extended follow-up [31, 32, 33].

This study has several important limitations that must be acknowledged. Firstly, the retrospective, non-randomised design introduces potential selection bias and confounding. Treatment allocation was based on clinical decision-making rather than randomisation. Although PSM was used to balance known baseline covariates, residual confounding from unmeasured factors (e.g., anxiety or depression severity, which were not formally assessed) cannot be excluded. Secondly, the concurrent use of eszopiclone in both groups makes it impossible to isolate the independent effect of guided imagery training; the observed benefits should be interpreted as the effect of the combined regimen. Thirdly, the single-centre design and relatively modest sample size limit the generalisability of the findings. Fourthly, the 4-week follow-up period is short, and the durability of the observed sleep improvements remains unknown. Finally, whilst adherence was monitored and reported, more detailed fidelity metrics for the guided imagery protocol were not available.

## Conclusions

In this retrospective cohort study of patients with COPD and sleep disorders, the addition of guided imagery training to standard pharmacotherapy was associated with significant improvements in objective sleep architecture and subjective sleep quality compared with pharmacotherapy alone. No significant changes in pulmonary function were observed. Whilst these findings are promising, they are constrained by the inherent limitations of the retrospective, non-randomised design and the confounding influence of concomitant eszopiclone use. Well-designed prospective, randomised controlled trials with longer follow-up are necessary to confirm these preliminary observations and definitively establish the role of guided imagery training as an adjunctive therapy for sleep disorders in COPD.

## Availability of Data and Materials

All experimental data included in this study can be obtained by contacting the corresponding authors if needed.
